# Host Plant Odour and Sex Pheromone are Integral to Mate Finding in Codling Moth

**DOI:** 10.1007/s10886-025-01568-4

**Published:** 2025-01-27

**Authors:** Anna Laura Erdei, Maria Sousa, Francisco Gonzalez, Marie Bengtsson, Peter Witzgall

**Affiliations:** 1 Department Plant Protection Biology, SLU Alnarp, Lomma, Sweden; 2 SLU, Kemisk Ekologi, Box 190, Lomma, SE-234 22 Sweden

**Keywords:** Mate recognition, Host plant choice, Reproductive isolation, Natural selection, Sexual selection, Sympatric speciation

## Abstract

The great diversity of specialist plant-feeding insects suggests that host plant shifts may initiate speciation, even without geographic barriers. Pheromones and kairomones mediate sexual communication and host choice, and the response to these behaviour-modifying chemicals is under sexual and natural selection, respectively. The concept that the interaction of mate signals and habitat cues facilitates reproductive isolation and ecological speciation is well established, while the traits and the underlying sensory mechanisms remain unknown. The larva of codling moth feeds in apple and other rosaceous fruits. We show for the first time that the response of male moths to female sex pheromone codlemone relies upon presence of pear ester, a kairomone emitted by host fruit. In the non-host tree birch, attraction to pheromone alone is very strongly reduced, but is fully rescued by blending pheromone with kairomone. This affords a mechanism how host plant shifts shape new mate-finding signals that can give rise to assortative mating and reproductive isolation.

## Introduction

The extraordinary diversity of plant-feeding insects arises from specific host plant associations and therefore depends on host plant recognition (Ehrlich and Raven 1964; Jaenike 1990; Nosil et al. 2002). Insects, and especially nocturnal insects, use first of all the sense of smell to find their plant hosts. Plant odorants mediate behavioural responses directed to the plant, like feeding and oviposition. And, plant odorants also contribute to specific mate communication, in concert with sex pheromones, when matings occur on the host plant (Smadja and Butlin 2009; Janz 2011; Borrero-Echeverry et al. 2018; Conchou et al. 2019; Jarrett and Miller 2024).

Taxonomically related insects are often found on taxonomically related plants, suggesting that host plant shifts contribute to reproductive isolation and phylogenetic divergence. In fact, mating preferences linked to the habitat or host plant are under combined sexual and natural selection, which is probably sufficient to initiate and complete speciation (Blows et al. 2002; Bolnick and Fitzpatrick 2007; Maan and Seehausen 2011; Butlin et al. 2012; Scordato et al. 2014; Rosenthal 2017).

The concept that host plant and mate choice are linked has long been established, while the underlying sensory and behavioural mechanisms are not yet resolved. The larvae of codling moth *Cydia pomonella* (Lepidoptera: Tortricidae) feed on apple and pear throughout their geographic range, and in some regions also on walnut, or on other rosaceous fruits, apricot, plum or quince (Bovey 1966; Barnes 1991). Larvae of its sibling species *C. pyrivora*, in comparison, are strictly monophagous on pear (Bovey 1966). Codling moth populations on apricot and walnut are ecologically and genetically differentiated from sympatric populations on apple (Bovey 1949; Cisneros and Barnes 1974; Pashley and Bush 1979; Thaler et al. 2008; Chen and Dorn 2010) and host phenology contributes to this differentiation of host populations (Bovey 1949, 1966; Philips & Barnes 1975).

Codling moth mating generally occurs on the host plant (Philips & Barnes 1975; Audemard 1991, Schumacher et al. 1997; Witzgall et al. 2005). During the diel flight period after sunset, female moths release sex pheromone to attract males. Females are often found in host trees with strong fruit setting and males are seen to swarm around the canopies of these trees already before the onset of female pheromone calling (Witzgall et al. 1996a, 1999; Bäckman et al. 1997). The main sex pheromone component is (*E*,* E*)-8,10-dodecadienol (codlemone) (Roelofs et al. 1971; Arn et al. 1985). The host plant odorant or kairomone ethyl (*E*,* Z*)-2,4-decadienoate (pear ester) attracts males and females, but male attraction to pear ester is weak compared to codlemone (Light et al. 2001; Light and Knight 2005; Knight et al. 2018).

Molecular and physiological evidence supports the idea that a configural blend of pheromone and kairomone is particularly powerful in codling moth. The kairomone pear ester is perceived via an olfactory receptor belonging to the clade of pheromone receptors (Bengtsson et al. 2014; Wan et al. 2019), which is reflected by the conspicuously synergistic response to a blend of codlemone and pear ester in the olfactory centre in the brain, the antennal lobe. In males, olfactory neurons tuned to codlemone and pear ester project to the area that is dedicated to processing female sex pheromone (Trona et al. 2010, 2013).

Field experiments with codling moth are only possible in orchards, where testing pear ester is compromised by interference with host plant odorants. We solved this dilemma by placing traps in adjacent apple and non-host birch trees to provide different background volatiles. Female sex pheromone by itself elicited strong male attraction only in apple. In birch, attraction to pheromone was strongly reduced, but was rescued by blending pheromone with pear ester. This experiment complements the physiological evidence and demonstrates the outstanding behavioural role of a blend of sex pheromone and kairomone, codlemone and pear ester.

Pear ester is characteristic for the aroma of ripe pear (Jennings et al. 1964), and has also been found in ripe apple (Berger et al. 1984). We asked whether occurrence of pear ester in green immature apples, which are suitable for codling moth oviposition and larval development, may have been overlooked. Males easily detect codlemone, which calling females emit at a few nanograms per hour (Bäckman et al. 1997), and they are expected to be equally sensitive to pear ester (Bengtsson et al. 2014). We therefore enhanced the chemical analysis of apple volatiles by using the olfactory receptor CpomOR3 as a most sensitive detector for pear ester (Bengtsson et al. 2014), and show that green apples do indeed release small amounts of pear ester.

Taken together, chemical and behavioural analysis establishes pear ester as a codling moth kairomone or host plant odorant. Moreover, our field test shows, for the first time, the interaction between a mate signal and a specific host plant cue. This provides a mechanistic understanding how shifts in either host plant association or sex pheromone composition can shape new mate-finding signals that give rise to reproductive isolation.

## Methods and Materials

### Chemicals and Trap Lures

Codlemone, (*E,E*)-8,10-dodecadienol (CAS 57002-06-9), a gift from K. Ogawa (Shin-Etsu Chemical Co., Chiyoda, Japan), was isomerically > 99.6% and chemically 99.9% pure. Chemical and isomeric purity of pear ester, ethyl (*E,Z*)-2,4-decadienoate (Sigma Aldrich, CAS 3035-30-7), was 98.2% and 95.6%, respectively. Compounds for field trapping were diluted in hexane (Sigma Aldrich, redistilled) and applied to 20-mm red serum bottle stoppers (Wheaton, VWR), which were used as field lures. Lures contained 100 µg codlemone, or 100 µg pear ester, or a blend of 100 µg codlemone and 100 µg pear ester.

(R)-(+)-Limonene (CAS 5989-27-5) and (S)-(-)-Limonene (CAS 5989-54-8) used as authentic standards were purchased from Sigma Aldrich. Reference compounds for single sensillum recordings were ethyl hexanoate (CAS 123-66-0, Sigma Aldrich), ethyl 3-hydroxybutyrate (CAS 5405-41-4, Acros), 2-heptanone (CAS 110-43-0, Fluka), (*E*)-2-hexenal (CAS 6728-26-3, Sigma Aldrich), 3-hydroxy-2-butanone (acetoin; CAS 513-86-0, Sigma Aldrich), DL-3-octanol (CAS 589-98-0, Acros) and pentyl acetate (CAS 628-63-7, Fluka).

### Field Trapping

Tetra traps (Arn et al. 1979) were placed in an apple orchard near Kivik (Scania, Sweden), where rows of apple trees (*Malus domestica*) are interspersed with windbreak rows of birch (*Betula pubescens*). Birch trees do not support development of codling moth larvae. Rows of apple are directly adjacent to the windbreak rows, on both sides, stem-to-stem distance between apple and birch is 5 m, branches of these trees were 2–3 m apart. Apple and birch trees were up to 5 m and 10 m tall, respectively. Twenty-two rows of apple are planted between single rows of birch, rows are 250 m long.

**Fig. 1:**
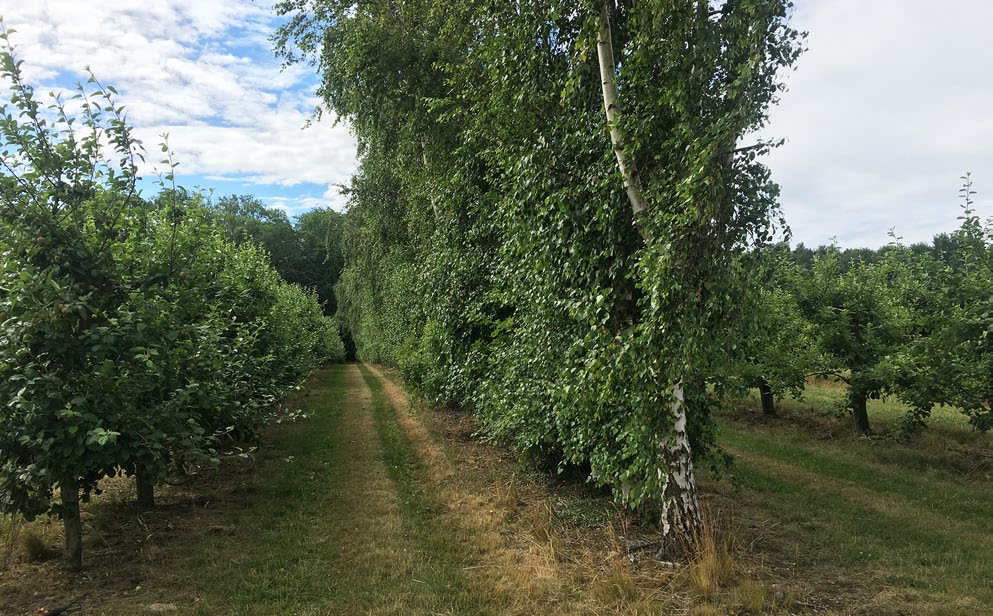
Field trapping site: apple orchard with birch windbreaks. Traps baited with synthetic codling moth pheromone, codlemone, the host-plant attractant (kairomone) pear ester, or a blend of both compounds were placed in an orchard, along rows of apple trees. The effect of volatiles of the host plant apple on male attraction was investigated by placing traps in adjacent windbreak rows of non-host birch trees. Stems of apple and birch trees were 5 m apart (Kivik, Scania, Sweden)

Traps were placed in rows of apple trees adjacent to birch, and also in rows of birch trees (Fig. 1). Traps placed along each row were ca. 10 m apart, and lines of 10 or 20 traps were placed along rows. Trap lines in apple and birch were spaced by 5 m. Traps were hung at ca. 2 m from the ground to green branches, checked every 2 to 3 d, and trap liners were exchanged. Trap colour was beige and blank traps did not capture moths. During 10 d, traps baited with codlemone were placed in apple (*n* = 30) and in birch (*n* = 30). After removing these traps, traps baited with pear ester (*n* = 50), and traps baited with the 1:1 blend of codlemone and pear ester (*n* = 50) were placed in apple and in birch, each, during 10 d. Traps baited with pear ester and the blend of codlemone and pear ester were alternated. The experimental orchard and surrounding apple orchards were not treated with pheromone dispensers for codling moth mating disruption. For statistical analysis, trap catch data in apple trees and in birch trees were log-transformed and compared using a two-tailed Student’s t-test (SPSS v. 29.0, IBM Corp.).

### Volatile Collections

Volatile collections were made from green fruiting branches of apple (cv. Aroma, with 2 or 3 green apples, up to 5 cm Ø, *n* = 8), from branches of birch with catkins (*Betula pubescens*, *n* = 7) and from apples (fruit only, 0.8 to 1 kg, cv. Aroma, *n* = 3). Plant material was sampled from trees in the orchard in Kivik, during the trapping experiment in 2023. For further analysis of pear ester, green apples (up to 5 cm Ø, cv. Aroma, *n* = 9) were sampled in the same orchard in 2024.

Plant material was brought to the laboratory, the cut ends of branches were held in 10-mL vials of water. It has been shown that apple branches released the same volatiles before and after they were cut, only the blend proportions differed (Bäckman et al. 2001). Branches or fruit were confined in 2-L glass containers. Ground-glass fittings were used to close the jar, and to connect air inlet and outlet. A charcoal-filtered airstream was pulled over the plant material from the bottom to the top of the jar, and through a 50-mg Super Q trap (80/100 mesh; Alltech, Deerfield, IL) held between plugs of glass-wool in a 4 × 40-mm glass tube. Before use, traps were kept in redistilled hexane for 15 min in an ultrasonic bath, and were then rinsed with 3 mL of methanol and redistilled hexane, successively. The air flow was 150 mL/min, exchanging the headspace in the jar 4.5 times/h. Collections were done during 24 h, at 20 to 22 °C and 10 to 30 lx. All glassware was heated to 375 °C for 8 h, before use. Volatile traps were extracted with 300 µL of redistilled hexane (LabScan, Malmö, Sweden) and sample volumes were then reduced to 60 to 80 µL at ambient temperature in Francke-vials with an elongated tip (5 cm x 2 mm i.d.) and stored in sealed glass capillary tubes at -18 °C.

### Chemical Analysis

For chemical analysis, samples were manually injected on a gas chromatograph coupled to a mass spectrometer (GC-MS; 7890 GC and 5977 MS, Agilent), with a DB-Wax fused silica capillary column (60 m x 250 μm x 0.25 μm; Agilent) operating in splitless mode. The GC oven temperature was programed from 30 °C (3 min hold) at 8 °C/min to 225 °C (10 min hold). Helium was used as carrier. Mass spectra were obtained from a Quadrupole mass selective detector, with an electron impact (EI) ionization at 70 eV in scan mode, from m/z 29 to 400. The temperature of the ion source was 230 °C. Agilent Mass Hunter (v 10.0) was used for analysis of GC-MS data. Compounds were tentatively identified by comparing mass spectra to libraries (NIST11 and Wiley12), using NIST MS Search (v. 2.4) and by comparing Kovats retention indices to NIST WebBook or PubChem databases. Compounds of particular interest were identified in comparison with synthetic standards, according to mass spectra and retention times.

For identification of pear ester in apple headspace, samples were also run on a GC coupled with a quadrupole-time of flight MS (GC-QTOF; 7890B GC and 7250 QTOF MS, Agilent) with HP-5 capillary column (60 m x 250 μm x 0.25 μm; J&W Scientific, Folsom, CA, USA). The inlet temperature was set to 280 °C and the oven temperature was increased from 40 °C (2 min hold) at 5 °C/min to 280 °C (12 min hold), the carrier gas was helium. The MS scanned 29 to 400 m/z units with an acquisition rate of 5 spectra/s, using 70 eV ionization energy.

For separation of chiral compounds, a Cyclodex-B fused silica capillary column (60 m x 250 μm x 0.25 μm; J&W) was used on a 6890 GC coupled to a 5975 MS (Agilent). Oven temperature was programmed from 35 °C (2 min hold) at 5 °C/min to 120 °C (8 min hold) and at 5 °C/min to to 220 °C. The enantiomer of limonene in birch volatile samples was identified following coelution of authentic standards of S-(-)-limonene and R-(+)-limonene.

Pear ester was identified, on both instruments (*n* = 3), according to its mass spectrum and retention index. The result was corroborated by coinjecting synthetic pear ester, in additional runs of the same samples. For quantification of pear ester in headspace collections, a dose-response curve was obtained by injecting known amounts of synthetic compound on the GC-MS (1, 10 and 25 ng; *n* = 3 to 4).

### Gas Chromatography Coupled with Electroantennogram Recordings

Headspace collections from apple and birch branches were screened with entire codling moth *C. pomonella* antennae (*n* = 5). Insects used for antennal recordings were lab-reared (Andermatt Biocontrol, Grossdietwil, Switzerland).

A HP 5890 GC, fitted with HP-INNOWax (30 m × 0.2 mm) or DB-Wax (30 m × 0.25 mm; J&W Scientific) capillary columns, programmed from 60 °C at 8 °C/min to 220 °C (10 min hold), was interfaced with an electroantennogram (EAG) apparatus for GC-coupled electroantennographic detection (GC-EAD; Arn et al. 1975).

The outlet of the GC-column was split 1:1 between the FID and a cut antenna of a *C. pomonella* male. The antenna was mounted in a holder, the cut ends were held in two wells containing Beadle–Ephrussi ringer solution. Compounds eluting from the capillary column were delivered to the antenna through a glass tube (120 × 8 mm) by a charcoal-filtered and humidified air stream. The EAG and FID signals were amplified and recorded simultaneously using GC-EAD software (v 4.6, Ockenfels Syntech GmbH, Buchenbach, Germany). EAG responses with a signal-to-noise ratio greater than 3.0 were considered significant. The EAG signal in response to compounds in headspace collections was normalized to the average response to a calibration stimulus, before and after the GC run, to account for differences between antennae and antennal preparations. The calibration stimulus was a 0.2-s puff from a Pasteur pipette, holding a filter paper (1 cm x 1 cm), formulated with 100 ng methyl salicylate in paraffin oil.

### Heterologous Expression of the *C. pomonella* Odorant Receptor CpomOR3 in *Drosophila melanogaster*

The protocol for cloning of CpomOR3 is found in Bengtsson et al. (2014) and Gonzalez et al. (2016). Following PCR amplification of CpomOR3, the purified product was cloned into the PCR8/GW/TOPO-TA plasmid (Invitrogen). The plasmid with the OR insert was then transferred to the destination vector pUASg-HA.attB (Bischof et al. 2007), using the Gateway LR Clonase II kit (Invitrogen) and the insert was checked by sequencing.

The pUAS-CpomOR3 transformation lines were generated by Best Gene (Chino Hills, CA, USA), using the PhiC31 genomic integration system (Bischof et al. 2007). Recombinant pUASg-HA.attB-CpomOR3 plasmids were injected into embryos of the #24749 Best Gene fly strain (genotype M{3xP3-RFP.attP}ZH-86Fb), containing an attP insertion locus for non-random integration on the third chromosome.

CpomOR3 was then expressed in the A neuron of ab3 basiconic sensilla (ab3A) through stepwise crossings with balancer lines according to Gonzalez et al. (2016). The final crossing was done with the *w; pOr22a-Gal4*^*ki*^*/CyO; TM2/TM6B* empty neuron line (Chahda et al. 2019) to produce flies of the genotype *w; pOr22a-Gal4*^*ki*^;*pUAS-CpomOR3* that were used for electrophysiological recordings.

### Single Sensillum Recordings

Following chemical analysis, the occurrence of pear ester in apple fruit headspace was corroborated by single sensillum (SSR) recordings, using *Drosophila melanogaster* flies expressing CpomOR3, the codling moth odorant receptor (OR) tuned to pear ester (Bengtsson et al. 2014).

Transgenic flies expressing Cpom3 were reared at 22 ± 2 °C on a sugar-yeast-cornmeal diet under a 12:12 L: D photoperiod and 3 to 6-d-old female flies were used for SSR. The flies were held in 100-µL pipette tips, with half of the head protruding and the ventral side facing upwards. The antennae were placed on a glass microscope slide and fixed between the 2nd and 3rd segment by a glass capillary that was held firm by dental wax. Antennal sensilla were localized under a light microscope at 1000x magnification (Olympus BX51W1, objective LMPLFLN). Charcoal-filtered, humidified air was blown over the preparation at 3 L/min via a glass tube (120 × 8 mm) that also delivered odorant stimuli.

Tungsten microelectrodes (Ø 0.12 mm, Harvard Apparatus Ltd, Edenbridge, UK) were electrolytically sharpened using graphite electrodes immersed in a saturated KNO_3_ solution. The recording electrode was positioned with a piezo-equipped (Märzhäuser PM-10) motor-controlled micromanipulator (Märzhäuser DC-3 K, Wetzlar, Germany) at the base of ab3 sensilla. The reference electrode was inserted in the eye. The signal from olfactory sensory neurons (OSNs) was amplified with a probe (INR-02; Syntech), digitally converted by an IDAC-4 (Syntech) interface, and analysed with Autospike v. 3.9 software (Ockenfels Syntech GmbH).

The glass tube delivering purified air to the preparation had an inlet (Ø 2 mm) for stimulation with puffs from pasteur pipette tips, holding filter papers with synthetic compound. In addition, the split outlet of a gas chromatograph (GC) was transferred through a capillary into this glass tube, for combined GC-SSR recordings. OSN signals were recorded continuously, either during entire GC runs or tests with synthetic compound, using Autospike software (v 3.8, Ockenfels Syntech GmbH). Responses of ab3A neurons, expressing CpomOR3, were calculated from changes in spike frequency, during 20 s before and after odorant stimulation, respectively. Spikes of the ab3B neuron, housed in the same sensillum, were disregarded; they are smaller in amplitude than ab3A neuron spikes.

For stimulation with synthetic odorants, 10 µL of diluted synthetic compounds (see below) was applied to filter paper (1 cm x 1 cm) held in glass pasteur pipettes. Puffs (2 mL air, during 0.5 s) from these pipettes, using a stimulus controller (Syntech SFC-1/b), were injected into the glass tube delivering an air stream to the preparation. A panel of odorants was composed to verify whether the recording electrode was correctly positioned in ab3 sensilla. Stimulation with ethyl 3-hydroxybutyrate (100 ng/µL), (*E*)-2-hexenal (10 ng/µL), 3-hydroxy-2-butanon (acetoin; 100 ng/µL) and pentyl acetate (100 ng/µL) served to exclude connection to other sensillum types. Contact with ab3 sensilla was then validated with 2-heptanone (100 ng/µL) and 3-octanol (100 ng/µL). And, stimulation with ethyl hexanoate (100 ng/µL) served to verify that the wild-type *Drosophila* OR was not expressed in ab3A. Acetoin was diluted in distilled water, all other compounds in paraffin oil. A dose response curve for synthetic pear ester (1, 10 and 25 ng; *n* = 3 to 4) was established using GC-SSR.

For GC-SSR recordings (*n* = 3), a 7890B GC (Agilent) was equipped with a DB-WAX UI capillary column (30 m x 250 μm x 0.25 μm, J&W Scientific). The hydrogen carrier effluent was split equally by a low dead-volume four-way cross connected to two deactivated fused silica capillary columns (0.32 mm x 0.25 μm) that were both 100 cm long. One capillary lead to the flame ionization detector (FID; 250 °C) and the other, through a Gerstel ODP-2 transfer line heated to 250 °C, into the glass tube with a 3-L/min air flow, for stimulus delivery to the antennal preparation. GC inlet temperature was set to 225 °C and the samples were injected in splitless mode. The oven was programmed from 40 °C at 20 °C/min to 225 °C (20 min hold).

## Results

**Fig. 2:**
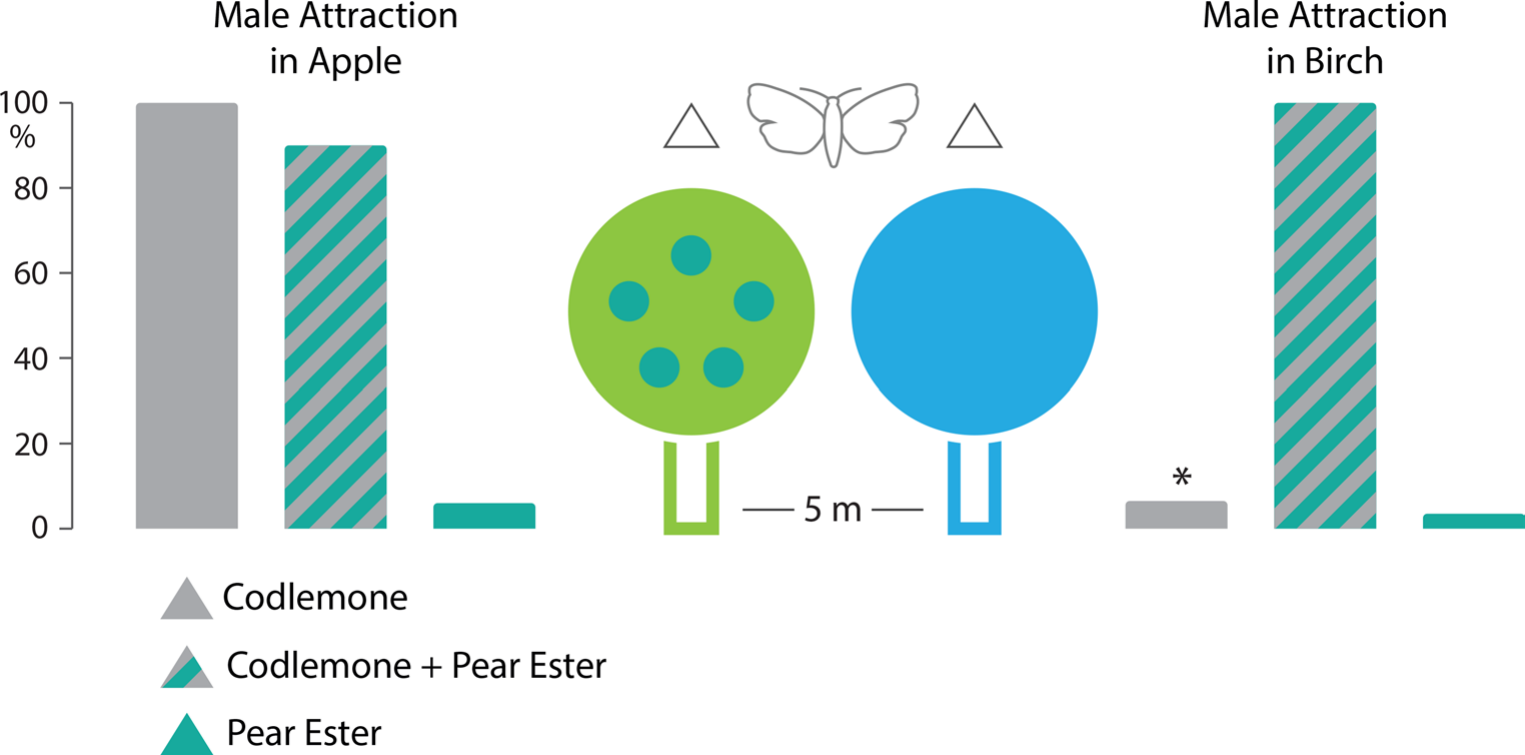
Male codling moth trap captures in apple and adjacent birch trees. Male moth attraction to 100 µg of sex pheromone codlemone was significantly higher in apple (10.7 ± 9.2 males/trap) than in birch (0.7 ± 1.2 males/trap; t = 7.05234, *p* < 0.00001, *n* = 30, two-tailed t-test). In a subsequent test, attraction to a blend of 100 µg sex pheromone codlemone and 100 µg of the kairomone pear ester was not significantly different in apple (7.3 ± 3.3 males/trap) and adjacent birch trees (8.1 ± 7.6 males/trap; t = 1.36183, *p* = 0.17637, *n* = 50, two-tailed t-test). Pear ester by itself is only a weak attractant, captures were 0.5 ± 0.9 and 0.3 ± 0.6 males/trap (*n* = 50) in apple and birch, respectively (t = 1.08671, *p* = 0.279831, two-tailed t-test)

We first asked whether a background of plant volatiles has an effect on male codling moth attraction to female sex pheromone, codlemone. In an apple orchard, traps were placed in apple trees and in directly adjacent birch trees. Pheromone traps captured 320 codling moth males (10.7 ± 9.2, *n* = 30) in apple and 20 males (0.7 ± 1.2, *n* = 30) in birch trees, even though apple trees and birch trees were only 5 m apart (Figs. 1 and 2). The difference in male attraction to sex pheromone in apple and in birch was significant (t = 7.05234, *p* < 0.00001, two-tailed t-test).

We next asked whether adding the kairomone (host plant attractant) pear ester to pheromone increases attraction in birch, compared with pheromone alone. Quite surprisingly, traps baited with a blend of codlemone and pear ester captured 365 males (7.3 ± 3.3, *n* = 50) in apple trees and 406 males (8.1 ± 7.6, *n* = 50) in birch trees (Fig. 2). The difference in captures with the blend lure in apple and in birch was not significant (t = 1.36183, *p* = 0.17637, two-tailed t-test).

Pear ester by itself is known to be a weak attractant (e.g. Knight et al. 2018), traps baited with pear ester captured 27 males (0.5 ± 0.9, *n* = 50) and 17 males (0.3 ± 0.6, *n* = 50) in apple and birch trees, respectively (Fig. 2; t = 1.08671, *p* = 0.279831, two-tailed t-test).

**Fig. 3:**
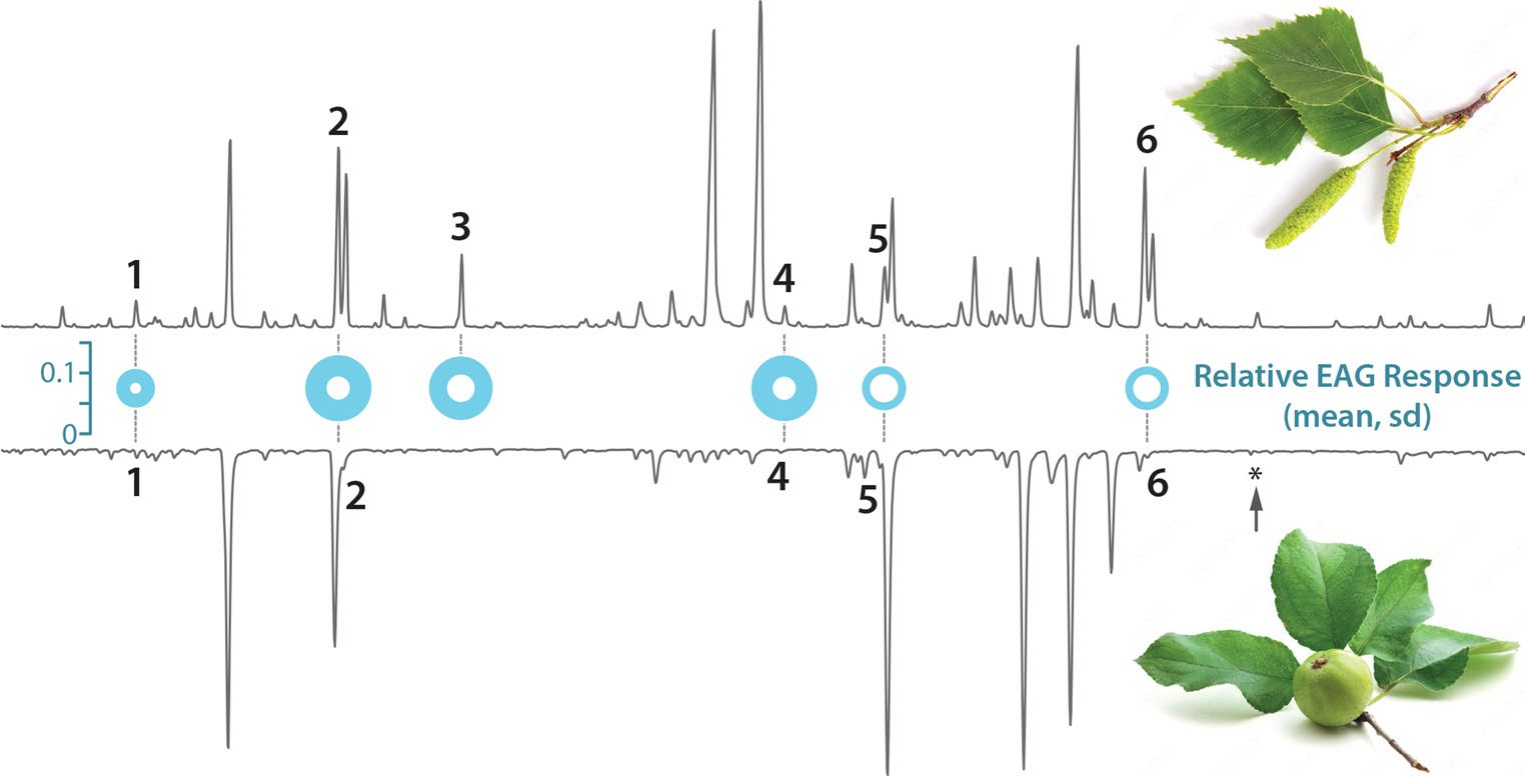
Volatiles eliciting an antennal response in codling moth males, in headspace collections of green fruiting branches of birch and apple (GC-EAD). Gas chromatograms (GC) of birch (top trace) and apple volatiles (bottom trace, inverted). Full circles show the mean electroantennographic (EAG) response to volatiles in birch headspace, relative to a standard stimulus; superimposed empty circles show the standard deviation (*n* = 5). Active compounds are S-(-)-limonene (1), (*E*)-4,8-dimethyl-1,3,7-nonatriene (DMNT) (2), (*E*)-β-ocimene (3), α-copaene (4), β-cubebene (5) and δ-cadinene (6). Dashed lines show co-occurrence of compounds in apple and birch headspace. The arrow shows the retention time of pear ester, which was below detection threshold in collections from apple branches

We concluded that the discrepancy of pheromone attraction in apple trees and non-host birch trees was due to presence or absence of volatiles in the odorant background surrounding these plants, either attraction synergists released from apple, or attraction antagonists from birch, or both. We therefore analysed volatile collections from birch and apple, collected from trees at the experimental site during the trapping experiment, by GC-MS (data available online). Birch volatiles were also screened for compounds eliciting antennal activity in codling moth males (Fig. 3), while antennal responses to apple volatiles have already been investigated (e.g. Bengtsson et al. 2001).

The birch volatiles that consistently elicited an antennal response in codling moth are all ubiquitous plant volatiles and they co-occured in apple headspace collected at the same site (Fig. 3), with the exception of (*E*)-β-ocimene. However, this compound has been found in apple in earlier studies (Bengtsson et al. 2001; Casado et al. 2006) and did not show an antagonistic effect on codling moth attraction (Knight et al. 2014).

The absence of attraction antagonists in birch (Fig. 3), together with a pronounced effect of pear ester on pheromone attraction in birch, but not in apple (Fig. 2), motivated a targeted search of pear ester in apple headspace, by chemical analysis (GC-MS and GC-QTOF) and single sensillum recordings (GC-SSR): the outlet of a GC column was screened with electrophysiological recordings from single sensilla expressing CpomOR3, a codling moth odorant receptor that is specifically tuned to pear ester.

First runs with headspace collected from green branches with only a few apple fruits did not contain pear ester at amounts above detection threshold (Fig. 3). Since codling moth females oviposit near green apples and since males are attracted to trees with fruit, we then examined fruit volatiles. Chemical analysis of apples collected in the experimental orchard, following the trapping experiment, confirmed presence of pear ester in fruit headspace, at a release rate of 0.013 ± 0.008 ng/kg apple/h (*n* = 3).

**Fig. 4:**
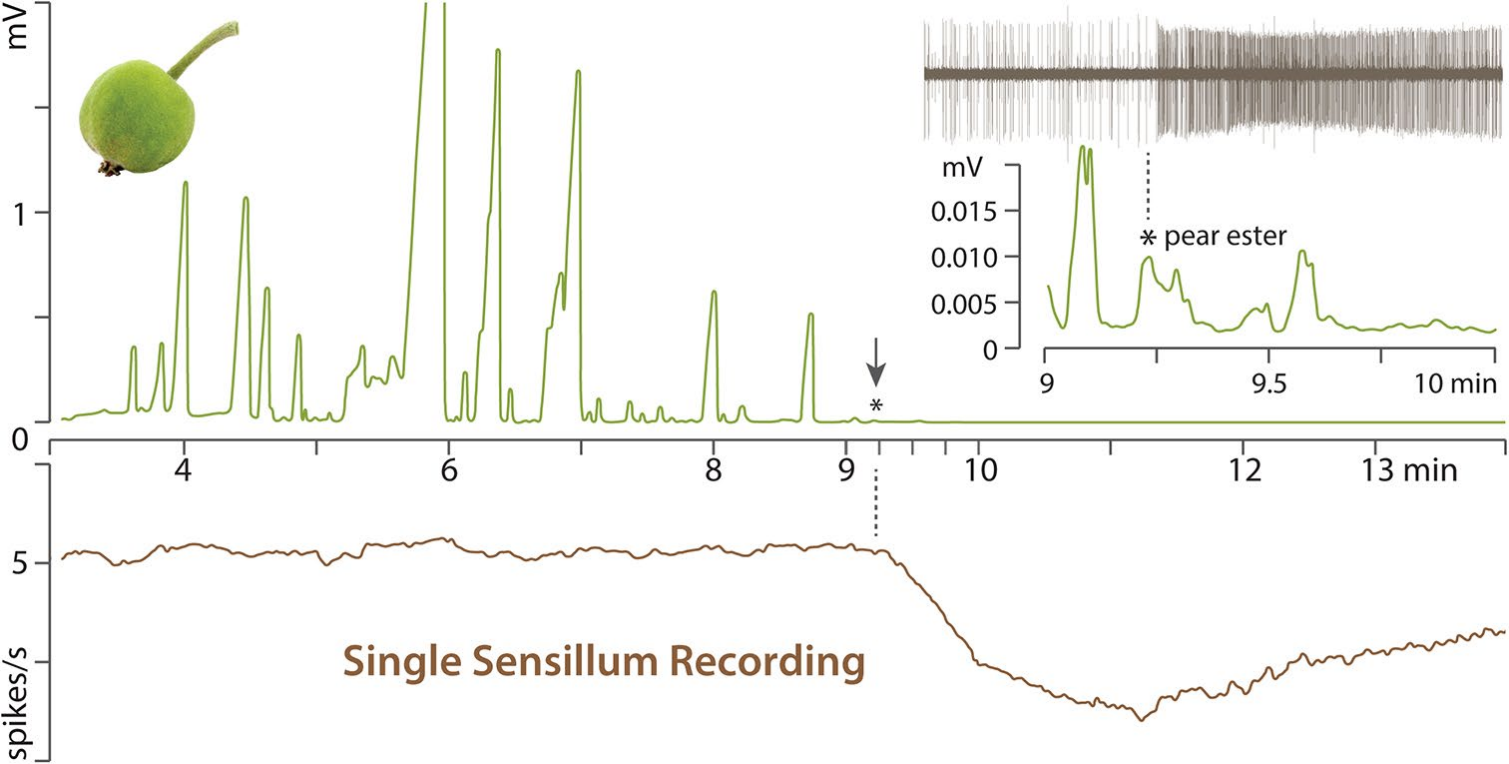
Pear ester detection in apple fruit headspace by gas chromatography coupled to single sensillum recordings (GC-SSR). Gas chromatogram (GC) of volatiles from green apple fruit (upper trace; mV) and corresponding response of a single olfactory sensory neuron (OSN) to the eluting compounds, as measured by single sensillum recordings (SSR) (lower trace; spikes/s). The GC column is split 1:1 between the flame ionization detector and a *Drosophila* antenna, where an electrode is inserted into an ab3 sensillum, housing an OSN that expresses the codling moth olfactory receptor CpomOR3, for which pear ester is the main ligand. Inset shows an amplified chromatogram, between 9 and 10 min, and a spike train recorded from a single OSN in response to pear ester, which elutes at 9.23 min

Pear ester was conclusively identified, in further volatiles collections from fruit, according to its mass spectrum and retention indices on two columns, and by coinjection of synthetic pear ester in separate runs of the same samples. Presence of pear ester in apple fruit headspace was finally corroborated by GC-SSR recordings (12.9 ± 4.1 spikes/s; *n* = 3), where antennal sensilla expressing CpomOR3 began to respond at the retention time of pear ester (Fig. 4).

Taken together, GC-MS and GC-SSR analysis confirms presence of pear ester as an apple volatile, which explains the result of our field trapping test (Fig. 2). Pear ester is present as a background odorant in apple but not in birch. In comparison, synthetic codlemone lures compete with codling moth females only during a part of the male diel flight period (Bäckman et al. 1997; Witzgall et al. 1999).

## Discussion

### Sex Pheromone and Host Odour Mediate Mate Finding in Codling Moth

We show that chemosensory attraction of codling moth males to females is mediated by a blend of sex pheromone and host plant odour, and that both are integral components of mate recognition and mate finding (Trona et al. 2013; Borrero-Echeverry et al. 2018; Jarrett and Miller 2024). It follows that divergence to new host plants or changes in pheromone biosynthetic pathways generate new premating communication channels that may lead to reproductive isolation.

Our field study provides experimental evidence for the concept that mating preferences coupled to habitat choice lead to an interaction between sexual and natural selection, which facilitates ecological divergence and speciation (Blows 2002; Bolnick and Fitzpatrick 2007; Ritchie 2007; Schluter 2009; Maan and Seehausen 2011; Servedio et al. 2011; Butlin et al. 2012; Debelle et al. 2014; Rosenthal 2017; Scordato et al. 2014).

We confirm that pear ester (Light et al. 2001; Light and Knight 2005) is a codling moth kairomone: according to its outstanding behavioural role, and its presence in the main host plant apple. This calls attention to a seemingly trivial point which has not received sufficient attention: in nature, sex pheromones are released into a background of plant odorants that includes kairomones. Codling moth male response to female sex pheromone and the kairomone pear ester cannot be disconnected, sexual signals and habitat cues are integral components of mate recognition. This makes it difficult or even obsolete to distinguish sexual from natural selection. A powerful interaction between insect and plant olfactory signals, in combination with a patchy distribution of host trees in natural habitats, will generate assortative mating and restrict gene flow even among populations and species inhabiting the same geographic region. The distinction of sympatry vs. allopatry does accordingly not apply to specialist herbivores where host choice is combined with mate choice (Mallet et al. 2009).

Field work with codling moth invariably shows that mating is restricted to host trees. In the laboratory, most field-collected insects fail to mate or oviposit in the absence of host plant odorants (Bovey 1966; Audemard 1991; Cisneros and Barnes 1974; Philips and Barnes 1975; Witzgall et al. 2005). Observations in orchards also show that females are not evenly distributed, but tend to aggregate around prominent trees with heavy fruiting. Males are seen flying around the crowns of these trees well before females release sex pheromone. They are easily attracted also to synthetic pheromone, codlemone, that elicits the full sequence of courtship behaviours. This makes traps baited with codlemone a valid proxy for attraction to females, even though calling females outcompete artificial pheromone sources (Bäckman et al. 1997; Witzgall et al. 1996a, 1999; El-Sayed et al. 1999; Bengtsson et al. 2001).

That male moths are on their wings and fly about their host plants, just before the onset of the diel mating period, has been observed in many other tortricid moths (Bradley et al. 1979) and further confirms that habitat and mate choice are linked, and that the male response to host plant volatiles is under sexual selection.

### Blends of Sex Pheromone and Host Plant Odorants Encode Species-Specificity

The combined response to sex pheromone and host plant volatiles is also under natural selection, as the host plant plays a prominent role in reproductive isolation. The few *Cydia* species that use the same sex pheromone are all found on taxonomically distant hosts (Witzgall et al. 1996b). Pear moth *C. pyrivora*, feeding on wild and cultivated pear (Rosaceae) and pea moth *C. nigricana*, feeding on peas and vetches (Fabaceae), both use the same sex pheromone, (*E,E*)-8,10-dodecadienyl acetate (codlemone acetate) (Witzgall et al. 1993; Makranczy et al. 1998). Distant plants, like pea and pear, release distinctive bouquets of volatiles (Witzgall et al. 2005; Thoming and Norli 2015).

Pear moth and codling moth, on the other hand, are sibling species that can reliably be identified only by male genitalia. They both feed on pear, their flight periods overlap, but they use different, mutually antagonistic pheromones. Codling moth attraction to its sex pheromone codlemone is strongly reduced in the presence of pear moth pheromone, codlemone acetate, and vice versa (Witzgall et al. 1996b, 2010). Codling moth males express an olfactory receptor that is specifically tuned to heterospecific pheromone (Bäckman et al. 2000; Walker et al. 2016; Cattaneo et al. 2017).

Codling moth larvae feed on apple and pear in Northern Europe, and on walnut and several other rosaceous fruits in Southern Europe and North America (Pettey 1925; Madsen and Borden 1954; Bovey 1966; Barnes 1991). While there is ample evidence for host-associated genetic differentiation (Pashley and Bush 1979; Thaler et al. 2008; Chen and Dorn 2010), there are no apparent pheromone dialects among host populations (Dumenil et al. 2014), possibly because the pheromone codlemone is an alcohol. Species-specific pheromones composed of geometric isomers of unsaturated acetates are common, in contrast to corresponding blends of alcohol isomers (Witzgall et al. 2010; El-Sayed 2025).

Host plant races of several other moths use specific isomer blends of acetates as sex pheromones, such as European corn borer *Ostrinia nubilalis* and larch budworm, *Zeiraphera diniana* (Priesner and Baltensweiler 1987; Pelozuelo et al. 2004; Malausa et al. 2005). Assortative mating between these host races does not rely solely on different pheromone signatures, a contributing role of the host plant has been shown in *Z. diniana*, where females attract males from within the same trees (Emelianov et al. 2001), which is consistent with our observations in codling moth. In cotton leafworm *Spodoptera littoralis*, attraction to heterospecific pheromone of the sibling species *S*. *litura* is greatly reduced in the presence of the host plant (Borrero et al. 2018).

### Pear Ester is a Codling Moth Kairomone

Plant odorants mediate or modulate most elements of lepidopteran reproductive physiology and behaviour, including feeding, sexual maturation, sex attraction, courtship and oviposition. However, plants release a wide range of volatiles and their abundance does not correlate with insect behaviour. Moreover, we lack efficient tools to reliably identify the active compounds (Gonzalez et al. 2020).

The effect of plant volatiles on male attraction to female sex pheromone has been studied, mainly in the laboratory, but seemingly conflicting, synergistic and antagonistic effects have been found (Landolt and Phillips 1997; Reddy and Guerrero 2004; Gadenne et al. 2016; Borrero-Echeverry et al. 2018; Conchou et al. 2019). This substantiates the difficulty in assigning behavioural roles to plant volatiles.

Our field trapping test, in combination with apple headspace analysis conclusively identifies pear ester as the codling moth kairomone and demonstrates its essential contribution to sexual communication. Pear ester is a known codling moth attractant and pheromone synergist (Light et al. 2001; Light and Knight 2005; Trona et al. 2013), but only the comparison of male attraction in host and non-host trees reveals its outstanding behavioural importance. In addition, a reinvestigation of apple fruit headspace confirms the presence of very small amounts of pear ester.

Very low release rates of codlemone by calling females (Bäckman et al. 1997), and of pear ester from green apples is a limitation only for chemical analysis, and not for codling moth males, because they possess specific olfactory receptors (Bengtsson et al. 2014; Walker et al. 2016). Specificity is a much more important criterion, pear ester has only been found in fruits of codling moth host plants (Jennings et al. 1964; Berger et al. 1984; Takeoka et al. 1992; Versini et al. 2012; Lopez et al. 2022).

### Molecular and Physiological Mechanism of Codlemone-Pear Ester Interaction

The neurophysiology and behavioural ecology of pheromone-plant volatile interactions has been thoroughly investigated in moths (Christensen and Hildebrand 2002; Namiki et al. 2008; Deisig et al. 2012; Pregitzer et al. 2013; Chaffiol et al. 2014). The identification of key, behaviourally active plant volatiles that are perceived via dedicated olfactory channels, is a main requirement for investigating the cognitive architecture of plant-pheromone interactions.

Pear ester is perceived via CpomOR3, an olfactory receptor belonging to the conserved clade of lepidopteran pheromone receptors (Bengtsson et al. 2014; Walker et al. 2016; Gonzalez et al. 2017). Male-biased pheromone receptors are otherwise tuned to sex pheromones, and general odorant receptors to environmental odors including plant volatiles (Yuvaraj et al. 2017; Fleischer et al. 2018; Robertson 2019; Bastin-Heline et al. 2019). A recent codling moth genome assembly reveals a duplication of CpomOR3. Both copies are tuned to pear ester, but they also respond to the sex pheromone codlemone, according to heterologous expression in frog eggs (Wan et al. 2019). This underscores the interconnection between pheromone and kairomone communication and that CpomOR3 encodes both host plant location and mate finding. Traits that combine ecological and sexual selection are particularly powerful during phylogenetic divergence (Servedio et al. 2011; Merrill et al. 2012; Safran et al. 2013).

The synergism between codlemone and pear ester depends on specific peripheral detection via olfactory receptors expressed in antennal sensory neurons, followed by processing of this signal in the antennal lobe, the first olfactory center in the insect brain. Intracellular recordings from neurons projecting to the antennal lobe and functional imaging show a very strong synergistic interaction upon stimulation with a blend of codlemone and pear ester, especially in the macroglomerular complex. This compartment of the olfactory centre is dedicated to processing pheromone input (Bäckman et al. 2000; Hansson and Anton 2000; Trona et al. 2010, 2013).

Taken together, pheromone and kairomone, codlemone and pear ester, are perceived via olfactory receptors and sensory neurons that branch to the same area of the codling moth olfactory centre. The morphology and physiology of pheromone and pear ester perception reflects the behavioural synergism revealed by our field experiment.

## Conclusion

The combined evidence substantiates a deep interaction between host plant and sex pheromone communication in codling moth. This interaction builds on two compounds, codlemone and pear ester, that yield a strong behavioural synergism and encode host preference and mate recognition. And, the cognitive olfactory architecture of pheromone and kairomone communication in codling moth is experimentally tractable, from phylogenetically related olfactory receptors to signal encoding in the brain and behavioural output.

An exciting direction for future research is whether the same compound, pear ester, encodes host recognition also in codling moths feeding on walnut, a non-rosaceous fruit. This relates to the question whether pear ester is indeed produced by the host plant or rather by associated microorganisms (Witzgall et al. 2012; Ljunggren et al. 2019).

Linking the key active compounds to chemosensory receptors and neural circuits that encode host preference and mate recognition, in codling moth host races or in cognate species, will help to build a scenario of how host plant shifts give rise to new communication channels in plant-feeding insects.

## Data Availability

The data of this study are shown in the manuscript. Raw data are available from the Dryad Data repository (10.5061/dryad.9ghx3ffrj)Reviewer URL: https://datadryad.org/stash/share/U-fvrPW49sHZpVnNPrPfUOsiaDg-EgfSvDP3oc_orcY.

## Code Availability

Not applicable, custom computer code or algorithms have not been used.
