# Correction: Host Plant Odour and Sex Pheromone Are Integral To Mate Finding in Codling Moth

**DOI:** 10.1007/s10886-025-01588-0

**Published:** 2025-03-12

**Authors:** Anna Laura Erdei, Maria Sousa, Francisco Gonzalez, Marie Bengtsson, Peter Witzgall

**Affiliations:** 1 Department Plant Protection Biology, SLU Alnarp, Lomma, Sweden; 2 SLU, Kemisk Ekologi, Box 190, Lomma, SE-234 22 Sweden

**Correction: Journal of Chemical Ecology (2025) 51:13**

10.1007/s10886-025-01568-4

The published article “Host Plant Odour and Sex Pheromone are Integral to Mate Finding in Codling Moth”, published on 27 January 2025 spotted several errors. One is the first subheader in Discussion now starts with “Sex Heromone…” instead of “Sex Pheromone”. Another one is the placement of Figs. 2 and 3. The text on page 6 below Fig. 3 could be moved up, between Fig. 2 and Fig. 3.

The original article has been corrected.

